# Use of digital technologies to promote sexual health in young adults: a narrative review

**DOI:** 10.1590/0034-7167-2023-0434

**Published:** 2025-06-20

**Authors:** Aliete Cristina Gomes Dias Pedrosa da Cunha-Oliveira, Sagrario Gómez Cantarino, Diana Gabriela Simões Marques dos Santos, Beatriz Filipa Pinto Oliveira, Bernardo Marinho, Carolina Dias Ribeiro, Filipe dos Santos Rodrigues, José Hermínio Gomes, Eva Patrícia da Silva Guilherme Menino, Renata Karina Reis

**Affiliations:** I Escola Superior de Enfermagem de Coimbra, Unidade de Investigação em Ciências da Saúde: Enfermagem (UICISA: E). Coimbra, Portugal; II Universidade de Coimbra, Centro de Estudos Interdisciplinares - CEIS20. Coimbra, Portugal; III Facultad de Terapia Ocupacional, Logopedia y Enfermería. Universidad de Castilla-La Mancha. CP. 45006. Toledo, Spain; IV Escola Superior de Saúde (ESSLei) do Politécnico de Leiria. Leiria, Portugal; V Universidade de São Paulo. Ribeirão Preto, São Paulo, Brazil

**Keywords:** Sexual Health, Young Adult, Digital Technology, Health Promotion, Nursing, Salud Sexual, Adulto Joven, Tecnología Digital, Promoción De La Salud, Enfermería

## Abstract

**Objective:**

To map the evidence in the literature on the use of digital technologies to promote sexual health in young adults.

**Methods:**

Narrative literature review. Data were extracted, analyzed, and synthesized by three independent reviewers.

**Results:**

After the selection process, four studies were included. The articles reveal that the use of digital technologies (mobile phones/applications) translates into increased knowledge and improved sexual attitudes and behaviors among young adults.

**Final considerations:**

The use of digital technologies promotes the sexual health of young adults, so these strategies should be implemented by health professionals.

## RESUMEN

**Objetivo:**

Identificar la evidencia en la literatura sobre el uso de las tecnologías digitales para promover la salud sexual de los adultos jóvenes.

**Métodos:**

Revisión narrativa de la literatura. Tres revisores independientes extrajeron, analizaron y sintetizaron los artículos y los resultados.

**Resultados:**

Mediante el proceso de selección, se incluyeron cuatro estudios. Los artículos muestran que el uso de tecnologías digitales (teléfonos móviles/ aplicaciones) se traduce en un aumento de los conocimientos, una mejora de las actitudes y del comportamiento sexual de los adultos jóvenes.

**Consideraciones finales:**

El uso de la tecnología digital favorece la salud sexual de los adultos jóvenes, por lo que estas estrategias deben ser aplicadas por los profesionales sanitarios.

## INTRODUCTION

The human immunodeficiency virus (HIV) is a public health issue affecting approximately 38.4 million people worldwide. Every day, 4,000 people become infected with HIV, including 1,100 young people aged 15 to 24 years. If current trends continue, 1.2 million people will be newly infected with HIV in 2025, which corresponds to three times more than the 2025 target of 370,000 new infections^(1)^.

In Portugal, the number of infections has continued to rise in recent years. In 2019, 778 new cases of HIV infection were diagnosed, which corresponds to a rate of 7.6 cases/100,000 inhabitants. The highest rate of new diagnoses was in the 25-29 age group^(2)^.

Educational technology has the potential to play a significant role in the promotion of sexual health among young adults, who are the largest users and most at risk of sexually transmitted infections (STIs). Although the World Health Organization defines youth or young adults as those aged 15-24 years ^(3)^, we decided to include people up to 29 years due to the lack of articles on this topic in the original population. Scientific evidence shows that the highest rates of STIs are found in the population aged 15-29 years. A prime example is the evolution in the number of new HIV cases, which accounted for around 50% of cases between 1983 and 2018^(2)^.

Young people aged 16 to 30 have the highest rate of mobile phone use. The access, speed, and low cost of the short message service (SMS) have led to a variety of health-related applications, including appointment, vaccination, and medication reminders, disease self-management, diagnostic testing and results, and health promotion interventions^(4)^.

Disparities in sexual health education are prominent and mainly affect more vulnerable groups. As a result, the use of technology to reach these marginalized populations could potentially invoke change^(5)^. Thus, investment in this area will be a strong contribution to achieving the Sustainable Development Goals (SDGs), namely “Reduced inequalities” (SDG 10) and “Good health and well-being” (SDG 3).

In a preliminary search of MEDLINE (via EBSCO) and PROSPERO, no review studies (published or ongoing) were identified in this area.

Therefore, the following review question was formulated: “What is the evidence on the use of digital technologies to promote sexual health in young adults?”

## OBJECTIVE

To map the scientific evidence on the use of digital technologies to promote sexual health in young adults.

## METHODS

A narrative literature review^(6)^ was conducted because it plays an important role in incorporating evidence into clinical practice, bringing together scientific knowledge on a specific topic.

### Inclusion and exclusion criteria

The inclusion and exclusion criteria were selected based on the Population, Intervention, Comparator, Outcomes, and study Design (PICOD) framework. The narrative literature review included studies that: a) regarding the population, were carried out in participants aged 15 to 29 years; b) regarding the intervention, addressed digital technologies, and c) regarding the outcomes, addressed sexual health promotion. As for the type of studies, primary studies with an experimental or quasi-experimental design were included.

### Search strategy

The search was carried out in several databases to obtain a more precise set of publications. The following keywords were used: (“Sexual Health”; “Young Adult”; “Digital Technology”; “Health Promotion”) in the Cumulative Index of Nursing and Allied Health Literature (CINAHL) Complete database via EBSCOhost, the Medical Literature Analysis and Retrieval System Online (MEDLINE) database via EBSCOhost, and Academic search complete database via EBSCOhost (Chart 1).

**Chart 1:** Search conducted in the CINAHL Complete database via EBSCOhost

S6	((S1 AND S2 AND S3 AND S4) NOT (Systematic review or literature review))	24
S5	(S1 AND S2 AND S3 AND S4)	26
S4	(“Health promotion” [Title/Abstract] OR “Disease prevention” [Title/Abstract] OR “Health education” [Title/Abstract] OR “Promotion Health” [Title/Abstract])[Title/Abstract])	47,205
S3	(“Digital technology” [Title/Abstract] OR “Virtual technologies” [Title/Abstract] OR “Technology” [Title/Abstract] OR “Robotic technology” [Title/Abstract] OR “web” [Title/Abstract] OR “computer” [Title/Abstract] OR “tablet” [Title/Abstract] OR “mobile phone” [Title/Abstract] OR “smartphone” [Title/Abstract] OR “internet” [Title/Abstract] OR “social media” [Title/Abstract])	267,794
S2	(“Young adult” [Title/Abstract] OR “Young People” [Title/Abstract] OR “Emerging Adult” [Title/Abstract] OR “College Student” [Title/Abstract]	37,037
S1	(“Sexual Health” [Title/Abstract] OR “Sex Education” [Title/Abstract])	12,229

### Study selection and data extraction

Studies were selected by three independent reviewers using the Rayyan collaborative tool. This platform allowed duplicates to be eliminated and titles, abstracts, and full texts to be analyzed blindly between reviewers. Data extraction was carried out in line with the review objective and questions. The following data were extracted: author, year, country, and outcomes of interest. Any disagreements between reviewers were resolved through discussion or with a fourth reviewer.

## RESULTS

**Figure 1:**
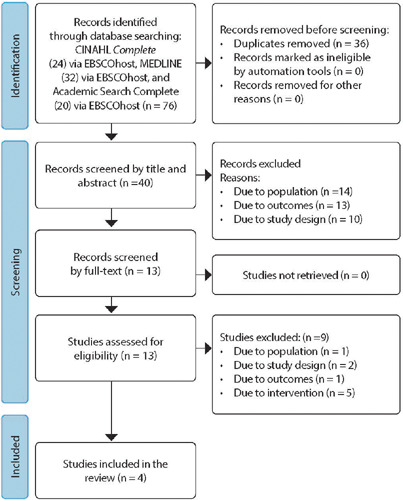
Flowchart of the study selection process

The search yielded 40 potentially relevant studies to answer the study question after duplicates were excluded from the 76 initial results. Of these 40 articles, 27 were excluded after title and abstract analysis, leaving 13 for full-text analysis (Figure 1).

Taking into account the review question and the inclusion and exclusion criteria, some articles were excluded: one study due to the population, two due to the study design, one due to the outcome of interest, and five due to the intervention. Therefore, four articles were included in the review (Chart 2).

**Chart 2:** Included studies

Code	Authors	Year	Country
**51** ^(4)^	Gold et al.	2011 a	Australia
**52** ^(7)^	Gold et al.	2011 b	Australia
**S3** ^(8)^	Lim et al.	2012	Australia
**S4** ^(9)^	Gannon et al.	2020	United States

Thus, the authors of S1, S2, and S3 considered knowledge, attitudes, and behaviors to be common outcomes.

As for the benefits of digital technology in promoting sexual health, the studies identified benefits in three areas: Knowledge, Attitudes, and Behaviors. We will therefore organize the discussion around these areas (Chart 3).

**Chart 3:** Synthesis of the data extracted from the included studies

Study	Benefits of digital technology in promoting sexual health among young adults
S1	- Increased knowledge in the experimental group; - Lower rates of multiple sexual partners; - Higher rate of condom use;
S2	- Increased knowledge in the experimental group; - Increased STI testing among women and men; - Higher rate of condom use;
S3	- Increased knowledge in the experimental group; - Increase in STI testing among women in the experimental group; - Increased health-seeking behaviors;
S4	- Effectiveness of the MyPEEPS app; - The usability of the health information technology was rated on a scale of 1-5, with a final score of more than 4 indicating high usability.

## DISCUSSION

Taking into account the results obtained through the synthesis of the included studies, the discussion is structured around three main topics: Knowledge, Attitudes, and Behaviors. These three topics reflect the domains in which digital technology operates: improving knowledge, attitudes, and behaviors related to sexual health.

### Knowledge

According to the authors of S1, a project was designed to send SMS messages to young adults in a city in Australia with sexual health education information. This study revealed a significant increase in young adults’ knowledge compared to baseline knowledge, demonstrating the effectiveness of SMS in sexual health education. Participants in the “sex” experimental group had significantly higher sexual health knowledge than those who received SMS messages about sun safety (odds ratio [OR] = 1.9; 95% confidence interval [CI] 1.0-3.8; *p* = 0.06)^(4)^.

With regard to S2, more than 55% (*n*=123) of men and more than 70% (*n*=268) of women answered correctly to 5 or all of the 6 questions on sexual health in the follow-up questionnaire. There was therefore a significant increase in correct answers compared to the baseline evaluation (*p* = 0.01), which also proved the effectiveness of the intervention^(7)^.

The authors of S3 also corroborated the hypothesis, showing with statistical data the increase in knowledge among young people. The level of knowledge was significantly higher in the experimental group than in the control group after 12 months of intervention in both female and male participants (Female - OR = 2.36; 95% CI [1.27 to 4.37]; Male - OR = 3.19; 95% CI [1.52 to 6.69])^(8)^.

Finally, with regard to S4, the authors showed favorable statistical data on the potential use of a mobile application for sexual health education. According to Gannon^(9)^, users rated the MyPEEPS app using the Post-Study System Usability Questionnaire (with scores ranging from 1 to 7, where 1 means *strongly agree* and 7 means *strongly disagree*). The scores fell below 2 on average, indicating high usability. Participants also rated the app using the Health Information Technology Usability Evaluation scale on a scale of 1-5 (where 1 means *strongly disagree* and 5 means *strongly agree*). The scores were higher than 4, indicating high usability.

### Attitudes

These articles also provided information on the attitudes of the participants.

Thus, the authors of S2 reported that around 23% (*n*=87) of women reported having had an STI test in the last 6 months at the follow-up, when compared to the baseline percentage of 18% (*n*=70). As for men, 10% (*n*=20) confirmed having had an STI test in the last 6 months at the follow-up, when compared to 8% (*n*=18) at the start of the study, thus indicating a significant increase in STI testing among men and women in the last 6 months^(7)^.

In line with the authors mentioned above, the S3 yielded positive results. At 12 months, female participants in the experimental group were significantly more likely to have had an STI test than women in the control group (OR = 2.51; 95% CI [1.11 to 5.69]).^(8)^ At 12 months, the proportion of young women in the control group (*n* = 131) who reported having been tested remained close to the community rate at 10%, while testing in the experimental group (*n* = 105) increased to 18%, suggesting that the intervention was the cause of the increase in testing^(8)^.

There were no differences in the proportions of male participants who reported having had an STI test between the experimental group and the control group (OR = 0.79; 95% CI [0.22 to 2.89])^(8)^.

In contrast, the authors of S1 found no significant differences between the groups in the frequency of STI testing (OR = 1.4, CI: 0.8-2.4, *p* = 0.25)^(4)^.

### Behaviors

In addition to their knowledge and attitudes, participants are also expected to demonstrate appropriate behaviors in the face of knowledge.

Thus, according to S2, a significant proportion of male participants reported a decrease in the number of multiple, casual, or new partners they had had during the study. This study also found that more than half of the respondents reported always using condoms with new partners compared to before^(7)^.

However, despite the significant increase in knowledge, S3 did not show a proportionally significant difference between the groups in terms of condom use with risky partners, in males, at any time point, with the data remaining similar to the start of the study (OR = 0.79 95% CI (0.22 to 2.89)^(8)^.

In S1, participants showed lower rates of multiple or new sexual partners and increased rates of condom use with new partners. Even so, there were no significant differences compared to the control group, and there was a potential increase in condom use and a reduction in the number of partners among the individuals in that group (*p* = 0.06)^(4)^.

Other studies corroborate these results, namely the study carried out by Costa et al.^(10)^. With another population of interest (adolescents), this study concluded that educational games, multimedia, and social networks are effective technologies in sexual health education among adolescents.

Sexual health education programs using digital technology can provide information and support to improve knowledge, attitudes, and behaviors related to sexual and reproductive health. Mobile health (mHealth) can promote the delivery of comprehensive, culturally relevant, and easily accessible sexual education - both of which are key facets to improving sexual health-related outcomes^(5)^ aligned with the SDGs “Reduced inequalities” (SDG 10) and “Good health and well-being” (SDG 3).

The four articles analyzed showed different limitations in their studies, such as the small number of questionnaires completed, resulting in insufficient statistical power (S1); the need for internet access during follow-up (S2); the loss to follow-up (S3); the sample included only individuals from urban areas, not generalizing the results to people living in rural and suburban areas.

### Study limitations

One limitation of this narrative review is that it should have included participants in the 15-24 age group, as well as experimental or quasi-experimental studies. Because the search for articles did not yield any studies with these participants, the age range had to be expanded. Another limitation was that it did not identify the state of the art on the subject, which is still underexplored.

### Contributions to the field

This literature review informs health professionals that digital technologies have facilitated sexual health promotion. They can use this knowledge in daily clinical practice to increase the sexual health literacy levels of their patients, namely young adults.

From a research perspective, it is important to carry out studies with an experimental or quasi-experimental design on this subject in Portugal, since the search did not yield any studies carried out in this country.

This study can reinforce the importance of investing in mHealth, particularly in the specific area of sexual health promotion.

In this way, we can guarantee the continuity of research-based knowledge, which will consequently increase the literacy of health professionals and the general population.

## CONCLUSION

The analysis of the scientific literature identified made it possible to conclude that the use of digital technologies, such as SMS, e-mail, and mobile applications, has facilitated sexual health promotion among young adults when comparing the experimental and control groups. Using digital technologies also led to a change in attitudes and behaviors, such as a significant increase in STI testing and an increase in health-seeking behaviors.

The use of SMS technology in the education of young people proved to facilitate the acquisition of knowledge. These results may be explained by its low cost, the interest of young people in using this technology daily as an activity they enjoy and through which they can acquire knowledge, and, finally, the rapid exchange of information between peers and friends with whom they are in daily contact through technology and, in turn, through text messaging.

Digital technologies are emerging as important health tools in modern health systems. Their development will lead to improved health indicators and, consequently, to a better quality of life, especially for young people.
