# Supplementary material for: Use of digital technologies to promote sexual health in young adults: a narrative review
Source: Rev Bras Enferm. 2025 Jun 20;78(Suppl 1):e20230434. doi: 10.1590/0034-7167-2023-0434 (PMC12185118; doi:10.1590/0034-7167-2023-0434)
Supplement: Translation

# Translation: O uso de tecnologias digitais na promoção da saúde sexual nos jovens adultos: revisão narrativa

**DOI:** 10.1590/0034-7167-2023-0434pt

**Authors:** Aliete Cristina Gomes Dias Pedrosa da Cunha-Oliveira, Sagrario Gómez Cantarino, Diana Gabriela Simões Marques dos Santos, Beatriz Filipa Pinto Oliveira, Bernardo Marinho, Carolina Dias Ribeiro, Filipe dos Santos Rodrigues, José Hermínio Gomes, Eva Patrícia da Silva Guilherme Menino, Renata Karina Reis

**Affiliations:** I Escola Superior de Enfermagem de Coimbra, Unidade de Investigação em Ciências da Saúde: Enfermagem (UICISA: E). Coimbra, Portugal; II Universidade de Coimbra, Centro de Estudos Interdisciplinares - CEIS20. Coimbra, Portugal; III Facultad de Terapia Ocupacional, Logopedia y Enfermería. Universidad de Castilla-La Mancha. C.P. 45006. Toledo, España; IV Escola Superior de Saúde (ESSLei) do Politécnico de Leiria. Leiria, Portugal; V Universidade de São Paulo. Ribeirão Preto, São Paulo, Brasil

**Keywords:** Saúde Sexual, Jovem Adulto, Tecnologia Digital, Promoção da Saúde, Enfermagem

## Abstract

**Objetivo:**

Identificar a evidência na literatura sobre o uso das tecnologias digitais na promoção da saúde sexual de jovens adultos.

**Método:**

Revisão narrativa da literatura. Três revisores independentes realizaram a extração, análise e síntese dos artigos e resultados.

**Resultados:**

Através do processo de seleção, quatro estudos foram incluídos. Os artigos revelam que o uso de tecnologias digitais (telemóvel/aplicações) se traduz num aumento do conhecimento e na melhoria das atitudes e dos comportamentos sexuais dos jovens adultos.

**Considerações finais:**

O uso da tecnologia digital promove a saúde sexual de jovens adultos, pelo que estas estratégias deverão ser implementadas pelos profissionais de saúde.

## INTRODUÇÃO

A Infeção por Vírus da imunodeficiência humana (VIH) é um problema de saúde pública e atinge aproximadamente 38,4 milhões de infetados em todo o mundo. Todos os dias, 4 mil pessoas — dentre elas, 1,1 mil jovens de 15 a 24 anos são infetadas. Se as tendências atuais continuarem, 1,2 milhões de pessoas serão infetadas pelo VIH em 2025 — três vezes mais do que a meta de 2025, que era de 370 mil novas infecções^(1)^.

Em Portugal, verificou-se um novo aumento de infeções nos últimos anos. Em 2019, foram diagnosticados 778 novos casos de infeção por VIH que equivale a uma taxa de 7,6 casos/100 mil habitantes. A taxa de novos diagnósticos mais elevada observou-se na faixa etária 25-29 anos^(2)^.

A tecnologia educacional tem um grande potencial para desempenhar um papel importante na promoção da saúde sexual dos jovens adultos, sendo estes os maiores utilizadores e os que correm maior risco de Infeções Sexualmente Transmissíveis (IST). A Organização Mundial da Saúde define a juventude ou os jovens adultos como a população que abrange a faixa etária dos 15 aos 24 anos^(3)^, contudo, considerou-se pertinente aumentar a população em estudo nos critérios de inclusão até aos 29 anos, devido à inexistência de artigos relacionados com esta temática na população inicialmente referida. A evidência científica refere que a população entre os 15 e os 29 anos tem um maior índice de transmissão de IST. Um exemplo paradigmático é a evolução do número de novos casos da Infecção por Vírus de Imunodeficiência Humana que representa cerca de 50% dos casos entre 1983 e 2018^(2)^.

Os jovens com idades compreendidas entre os 16 e os 30 anos são os que têm a taxa mais elevada de utilização de telemóveis. O acesso, a rapidez e o baixo custo dos SMS conduziram a uma variedade de aplicações relacionadas com a saúde, incluindo lembretes de consultas, lembretes de vacinação, medicação, autogestão de doenças, testes e resultados de diagnósticos e intervenções de promoção da saúde^(4)^.

As disparidades na educação em saúde sexual são proeminentes e afetam principalmente grupos mais vulneráveis. Como resultado, a utilização da tecnologia para alcançar estas populações marginalizadas poderia potencialmente invocar mudanças^(5)^. Assim, o investimento nesta área será um forte contributo considerando os Objetivos de Desenvolvimento Sustentável (ODS), nomeadamente para “Reduzir as desigualdades” (ODS 10) e para promover uma “Saúde de qualidade” (ODS 3).

Numa pesquisa preliminar realizada na MEDLINE (via EBSCO) e PROSPERO, não se identificaram estudos de revisão (publicada ou a ser realizada), neste âmbito.

Desta forma elaborou-se a questão de revisão: “Qual a evidência sobre o uso das tecnologias digitais na promoção da saúde sexual nos jovens adultos?”.

## OBJETIVO

Identificar a evidência científica sobre o uso das tecnologias digitais na promoção da saúde sexual de jovens adultos.

## MÉTODOS

Optou-se por realizar uma revisão narrativa da literatura^(6)^, visto que esta desempenha um papel importante na incorporação de evidências na prática clínica, reunindo conhecimento científico sobre um tema específico.

### Critérios de inclusão e exclusão

Os critérios de inclusão e exclusão foram selecionados com base nos elementos da população, intervenção, comparador, outcomes e desenho do estudo (PICOD). Utilizando estes elementos, foram incluídos na revisão narrativa da literatura estudos que: a) quanto ao tipo de participantes, sejam de idades compreendidas entre os 15 e os 29 anos; b) quanto à intervenção, abordem as tecnologias digitais, c) quanto ao outcomes, abordem a promoção da saúde sexual. Quanto ao tipo de estudos, foram incluídos estudos primários com desenho experimental ou quasi-experimental;

### Estratégia de pesquisa

A estratégia de pesquisa foi realizada em várias bases de dados distintas para se obter um conjunto mais preciso de publicações. Foram utilizadas as seguintes palavras-chaves (“Sexual Health”; “Young Adult”; “Digital Technology”; “Health Promotion”) nas bases de dados Cumulative Index to Nursing and Allied Health Literature (CINAHL) complete via EBSCO host, Medical Literature Analysis and Retrieval System Online (MEDLINE) via EBSCO host e Academic search complete via EBSCO host (Quadro 1).

**Quadro 1:** Pesquisa realizada na base de dados CINAHL complete via EBSCO host

S6	((S1 AND S2 AND S3 AND S4) NOT (Systematic review or literature review))	24
S5	(S1 AND S2 AND S3 AND S4)	26
S4	(“Health promotion” [Title/Abstract] OR “Disease prevention” [Title/Abstract] OR “Health education” [Title/Abstract] OR “Promotion Health” [Title/Abstract])[Title/Abstract])	47,205
S3	(“Digital technology” [Title/Abstract] OR “Virtual technologies” [Title/Abstract] OR “Technology” [Title/Abstract] OR “Robotic technology” [Title/Abstract] OR “web” [Title/Abstract] OR “computer” [Title/Abstract] OR “tablet” [Title/Abstract] OR “mobile phone” [Title/Abstract] OR “smartphone” [Title/Abstract] OR “internet” [Title/Abstract] OR “social media” [Title/Abstract])	267,794
S2	(“Young adult” [Title/Abstract] OR “Young People” [Title/Abstract] OR “Emerging Adult” [Title/Abstract] OR “College Student” [Title/Abstract]	37,037
S1	(“Sexual Health” [Title/Abstract] OR “Sex Education” [Title/Abstract])	12,229

### Critérios de inclusão e exclusão

Os critérios de inclusão e exclusão foram selecionados com base nos elementos da população, intervenção, comparador, outcomes e desenho do estudo (PICOD). Utilizando estes elementos, foram incluídos na revisão narrativa da literatura estudos que: a) quanto ao tipo de participantes, sejam de idades compreendidas entre os 15 e os 29 anos; b) quanto à intervenção, abordem as tecnologias digitais, c) quanto ao outcomes, abordem a promoção da saúde sexual. Quanto ao tipo de estudos, foram incluídos estudos primários com desenho experimental ou quasi-experimental;

### Estratégia de pesquisa

A estratégia de pesquisa foi realizada em várias bases de dados distintas para se obter um conjunto mais preciso de publicações. Foram utilizadas as seguintes palavras-chaves (“Sexual Health”; “Young Adult”; “Digital Technology”; “Health Promotion”) nas bases de dados Cumulative Index to Nursing and Allied Health Literature (CINAHL) complete via EBSCO host, Medical Literature Analysis and Retrieval System Online (MEDLINE) via EBSCO host e Academic search complete via EBSCO host.

### Seleção de estudos e extração de dados

A seleção de estudos foi realizada por três revisores independentes com uso de uma ferramenta de leitura colaborativa Rayyan. Esta plataforma permitiu a eliminação dos duplicados e a análise dos títulos, resumos e textos completos de forma oculta entre revisores. A extração de dados foi realizada de forma alinhada com o objetivo e questões de revisão. Os dados extraídos foram os seguintes: autor, ano, país e resultados de interesse. Desacordos entre os revisores foram resolvidos através de discussão ou com um quarto revisor.

## RESULTADOS

A busca resultou em 40 estudos potencialmente relevantes para responder a pergunta do estudo, após a eliminação dos duplicados (inicialmente, 76 resultados). Destes 40 artigos, foram excluídos 27 artigos após a leitura do título e o resumo e, consequentemente, selecionados 13 para leitura do texto integral (Figura 1).

**Figura 1:** Fluxograma do processo de seleção dos estudos

Relativamente aos últimos artigos mencionados, 1 estudo foi eliminado pelo critério utilizado para a população, 2 foram eliminados pelo desenho de estudo, 1 estudo foi eliminado pelo resultado e 5 estudos foram removidos pela intervenção, tendo em conta a questão de revisão e os critérios de inclusão e exclusão supramencionados. Considerando o já referido, obteve-se uma seleção final de 4 artigos (Quadro 2).

**Quadro 2:** Estudos selecionados

Codificação	Autores	Ano	País
**E1** ^(4)^	Gold, et al.	2011 a	Austrália
**E2** ^(7)^	Gold, et al.	2011 b	Austrália
**E3** ^(8)^	Lim, et al.	2012	Austrália
**E4** ^(9)^	Gannon, et al.	2020	Estados Unidos

Desta forma, os autores dos artigos (E1, E2 e E3) consideraram como *outcomes* comuns o conhecimento, as atitudes e os comportamentos.

Quanto aos benefícios da tecnologia digital na promoção da saúde sexual, os estudos obtidos, identificaram ganhos em três domínios: dos conhecimentos, das atitudes e dos comportamentos. Assim, iremos organizar a discussão em torno destes domínios (Quadro 3).

**Quadro 3:** Síntese da evidência extraída dos estudos incluídos

Estudo	Benefícios da tecnologia digital na promoção da saúde sexual dos jovens adultos
E1	- Aumento do conhecimento no grupo experimental; - Menores taxas de múltiplos parceiros sexuais; - Maior taxa de uso do preservativo;
E2	- Aumento do conhecimento no grupo experimental; - Aumento da testagem serológica às IST em ambos os sexos; - Maior taxa de uso do preservativo;
E3	- Aumento do conhecimento no grupo experimental; - Aumento da testagem serológica às IST nas mulheres do grupo experimental; - Aumento da procura de cuidados de saúde;
E4	- Efetividade da aplicação MyPEEPS; - A aplicação foi avaliada relativamente à usabilidade da tecnologia da informação em saúde, numa escala de 1-5, com classificação final superior a 4 o que indica alta usabilidade da mesma.

## DISCUSSÃO

Tendo em consideração os resultados obtidos através da síntese dos estudos incluídos, a discussão estrutura-se em três tópicos principais: Conhecimento, Atitudes e Comportamentos. Estes três tópicos refletem os domínios em que a tecnologia digital atua: melhoria de conhecimento, atitudes e comportamentos relacionados com a saúde sexual.

### Conhecimento

Segundo os autores do artigo E1, com a criação de um projeto que visava enviar *SMS* a jovens adultos, numa cidade da Austrália, com informações educativas sobre saúde sexual, este revelou posteriormente um crescimento significativo dos conhecimentos dos mesmos face ao conhecimento inicial, comprovando assim a efetividade do uso das mensagens na educação sexual. Os participantes pertencentes ao grupo experimental “sexo” tinham conhecimentos sobre saúde sexual significativamente mais elevados do que aqueles que receberam as mensagens sobre a proteção solar (odds ratio = 1,9; intervalo de confiança de 95% 1,0-3,8; p = 0,06)^(4)^.

No que concerne ao artigo E2, no questionário de follow-up realizado, mais de 55% (n=123) dos homens e mais de 70% (n = 268) das mulheres responderam corretamente a 5 ou mais das 6 perguntas sobre saúde sexual. Assim sendo, em comparação ao questionário inicial houve um aumento significativo nas respostas corretas (p = 0,01), pelo que o artigo comprovou também a efetividade da intervenção^(7)^.

Quanto ao artigo E3, o mesmo corrobora também a hipótese, mostrando com dados estatísticos o aumento do conhecimento nos jovens, sendo que o conhecimento do grupo experimental foi significativamente maior comparativamente ao grupo de controlo após 12 meses de intervenção em ambos os sexos (Sexo feminino - Odds ratio = 2,36; intervalo de confiança 95% (1,27 a 4,37); Sexo masculino - Odds ratio = 3,19; intervalo de confiança 95% (1,52 a 6,69)^(8)^.

Por fim, relativamente ao E4, os autores evidenciaram dados estatísticos favoráveis sobre o que foi delineado inicialmente quanto ao uso de uma aplicação móvel para educação sexual. Segundo Gannon^(9)^, os usuários da aplicação MyPEEPS utilizaram o Questionário de Usabilidade de Sistema Pós-estudo (com pontuações que variam entre 1 e 7, em que 1 significa concordo totalmente e 7 discordo totalmente) para classificar a aplicação, na qual resultou uma média abaixo de 2, o que indicou uma alta usabilidade por parte da mesma. Os participantes classificaram a aplicação também com a escala de avaliação de usabilidade de tecnologia da informação em saúde, numa escala de 1-5 (em que 1 significa discordo totalmente e 5 significa concordo totalmente), esta foi superior a 4 o que indica alta usabilidade da mesma.

### Atitudes

Face aos resultados dos artigos referidos, estes ofereceram também informações relativamente às atitudes tomadas por parte dos participantes.

Assim, os autores dos artigos E2 referiram que cerca de 23% (n=87) das mulheres relataram ter feito um teste serológico às IST nos últimos 6 meses no acompanhamento da intervenção, em comparação ao valor inicial de 18% (n=70). Quanto aos homens, 10% (n=20) confirmaram terem feito um teste serológico às IST nos últimos 6 meses no acompanhamento do estudo, em comparação com 8% (n=18) no início do estudo, indicando assim um aumento significativo na testagem às IST entre homens e mulheres nos últimos 6 meses^(7)^.

Em concordância com os autores acima, o estudo E3 alinhou-se com resultados positivos, aos 12 meses, as participantes do sexo feminino no grupo experimental eram significativamente mais suscetíveis de terem feito um teste serológico às IST do que as mulheres do grupo de controlo (Odds ratio = 2,51; intervalo de confiança 95% (1,11 a 5,69))^(8)^.

Ao fim desses 12 meses a proporção de jovens mulheres no grupo de controlo (n = 131) que relataram fazer um teste manteve-se próxima da taxa comunitária, em 10%, enquanto testes no grupo experimental (n = 105) aumentaram para 18%, sugerindo que a intervenção terá sido a causa do aumento da testagem^(8)^.

Não se registaram diferenças nas proporções de participantes do sexo masculino que relataram ter feito o teste serológico às IST entre o grupo experimental e o grupo de controlo (Odds ratio = 0,79; intervalo de confiança 95% (0,22 a 2,89))^(8)^.

Em contrapartida, os autores do estudo E1 não registaram diferenças significativas entre os grupos na frequência na realização de testes sorológicos às IST (Odds ratio = 1,4, intervalo de confiança: 0,8-2,4, p = 0,25)^(4)^.

### Comportamentos

Aliado ao conhecimento demonstrado pelos participantes dos estudos e também com as atitudes referidas, espera-se, consequentemente, que os mesmos demonstrem também comportamentos adequados face ao conhecimento.

Assim, segundo o artigo E2, uma proporção significativa dos homens relatou um decréscimo no que diz respeito aos múltiplos parceiros, casuais ou novos que tiveram ao longo do acompanhamento do estudo. Neste estudo, verificou-se também que mais de metade dos inquiridos referiram utilizar sempre preservativo quando estão com novos parceiros face a outrora^(7)^.

Por sua vez, apesar do significativo aumento do conhecimento, o estudo E3 não comprovou uma diferença proporcionalmente significativa entre os grupos relativamente à utilização de preservativo com parceiros de risco, no sexo masculino, em qualquer momento, tendo mantido os dados semelhantes aquando do início do estudo (Odds ratio = 0,79 intervalo de confiança 95% (0,22 a 2,89))^(8)^.

Quanto ao estudo E1, este registou que os participantes mostraram menores taxas de múltiplos parceiros sexuais ou novos parceiros, bem como a taxa de utilizadores de preservativo com novos parceiros aumentou ao mesmo tempo. Ainda assim, face ao grupo de controlo, não se registaram diferenças significativas, tendo havido possivelmente uma melhoria da utilização de preservativos e redução no número de parceiros por parte dos indivíduos desse grupo (p = 0,06).^(4)^

Outros estudos corroboram estes resultados, nomeadamente o estudo desenvolvido por Costa et al.^(10)^, com outra população de interesse (adolescentes) concluiu que os jogos educativos, as multimídias e as redes sociais são as tecnologias consideradas educativas e efetivas no processo de aprendizagem sobre educação sexual em adolescentes.

De facto os programas de educação em saúde sexual com recurso a tecnologia digital podem fornecer informações e apoio para melhorar o conhecimento, as atitudes e os comportamentos relacionados com a saúde sexual e reprodutiva. A educação sexual baseada em tecnologia digital móvel (mHealth) tem o potencial de fomentar a promoção da educação sexual abrangente, culturalmente relevante e facilmente acessível – sendo que ambas são facetas fundamentais para melhorar os resultados relacionados com a saúde sexual^(5)^, indo ao encontro dos ODS “Reduzir as desigualdades” (ODS 10) e promover uma “Saúde de qualidade” (ODS 3).

Os quatro artigos analisados mostraram diferentes limitações nos seus estudos como o número reduzido de questionários preenchidos, resultando assim na insuficiência estatística (E1); exigência de acesso à internet no acompanhamento (E2); perda do follow-up (E3); a amostra inclui apenas indivíduos de zonas urbanas, não generalizando os resultados para pessoas que vivem em áreas rurais e suburbanas.

### Limitações do Estudo

No que concerne às limitações da Revisão narrativa, o processo de pesquisa de artigos cuja população deveria corresponder à faixa etária dos 15 aos 24 anos bem como fossem estudos experimentais ou quasi-experimentais de tal forma que houve necessidade de aumentar o intervalo da população. Outro constrangimento sentido, passou por encontrar o estado da arte sobre a temática em estudo, visto que ainda é um tema pouco abordado.

### Contribuições para a área

Esta revisão da literatura permite informar os profissionais de saúde que através das tecnologias digitais há um aumento na promoção da saúde sexual, podendo transpor estes conhecimentos na sua prática clínica diária, aumentando assim a literacia em saúde sexual dos seus utentes, neste caso os jovens adultos.

Também, na ótica da investigação, consideramos importante a realização de artigos com desenho do estudo experimental ou quasi-experimental desta temática em Portugal, uma vez que na pesquisa efetuada não se obteve resultados neste país.

O presente estudo poderá reforçar a importância de se investir na saúde digital, nomeadamente nesta área específica para a promoção da saúde sexual.

Desta forma, podemos garantir a continuidade do conhecimento na investigação que, consequentemente, aumentará a literacia dos profissionais de saúde, bem como da população.

## CONSIDERAÇÕES FINAIS

Durante a análise da literatura científica indentificada foi possível concluir com clareza que o uso das tecnologias digitais permitiu um aumento da promoção da saúde sexual dos intervenientes visto que, houve um aumento ao nível do conhecimento, com o uso de tecnologias digitais como os SMS, E-mail e aplicações móveis quando comparados os grupos experimental e os grupos de controlo. O uso destas tecnologias digitais também permitiu uma mudança de atitudes e comportamentos como, por exemplo, o aumento significativo na testagem serológica às IST e um aumento na procura de cuidados de saúde.

Concluiu-se que o uso das mensagens enquanto tecnologia e como forma de educar os jovens mostrou ser, de facto, um procedimento favorável para a aquisição de conhecimento. Alguns prováveis fatores que podem explicar estes resultados são o custo reduzido, o interesse dos jovens no uso da tecnologia sendo esta uma prática diária onde podem aliar uma atividade que gostam à aquisição de conhecimento e, por fim, a rápida partilha da informação entre pares e amigos, uma vez que estes se conectam através da tecnologia e, por sua vez, por mensagens diariamente.

As tecnologias digitais emergem como instrumentos de saúde de relevo aplicados aos sistemas de saúde modernos. O seu desenvolvimento, conduz à melhoria de indicadores de saúde, nomeadamente dos jovens e consequentemente à melhoria da sua qualidade de vida.
